# Oleosines in sesame allergy

**DOI:** 10.1186/2045-7022-3-S3-P170

**Published:** 2013-07-25

**Authors:** PM Ojeda, I Ojeda, G Rubio, F Pineda

**Affiliations:** 1 Allergy, Clínica de Asma y Alergia Dres. Ojeda, Madrid, Spain; 2 Research, Diater Laboratories, Madrid, Spain

## Background

Although infrequent, sesame allergy is well known. Most of the cases are due to sensitization to water-soluble proteins. However, lipophilic proteins may be important [1].

## Methods

We present the case of a 31 y.o. woman with repeated anaphylactic reactions most of them upon eating at oriental restaurants. A vast allergic study was carried out in another center ruling out allergy/intolerance to several food additives and foods. The key fact for the diagnosis was an anaphylactic reaction upon eating home-made humus made of chickpea, olive oil and sesame oil. We performed wide skin prick testing (SPT) and specific IgE (both CAP and ISAC) for nuts, legumes, and sesame seed (commercial extract, natural raw sesame seed) as well as prick test, prick-prick, and prick patch with sesame oil, and open food challenge test (OFCT) with sesame seed.

## Results

SPT with foods including sesame seed: negative. Skin prick-patch test with sesame oil: positive. SDS-PAGE/immunoblotting: no IgE fixation against water soluble sesame extract proteins; IgE fixation to a 17-kDa protein from the lipidic sesame extract. This protein was identified as an oleosin (Ses i 4). OFCT positive with 2 grams of sesame seeds.

## Conclusion

Oleosins have been described by others [2], and are important allergens from sesame seed. Since they are hydrophobic, they are not present at commercial extracts or extract prepared from sesame seed in saline, or the CAP system extract. ISAC only contains Ses i 1. Prick test has to be performed with prick-patch test with immediate reading (15-20 min) [3]. The lipidic part of the seed has to be considered when studying unexplained cases of suspected sesame allergy.

## Disclosure of interest

None declared.
